# Gender Particularities and Prevalence of Atypical Clinical Presentation in Non-ST Elevation Acute Coronary Syndrome

**DOI:** 10.3390/jcdd9030084

**Published:** 2022-03-14

**Authors:** Mihai Octavian Negrea, Dumitru Zdrenghea, Minodora Teodoru, Bogdan Neamțu, Călin Remus Cipăian, Dana Pop

**Affiliations:** 1 Faculty of Medicine, “Lucian Blaga” University, 550024 Sibiu, Romania; dr.mihai.negrea@gmail.com (M.O.N.); calin.cipaian@ulbsibiu.ro (C.R.C.); 2 Fifth Department of Internal Medicine, Cardiology Rehabilitation, “Iuliu Hatieganu” University of Medicine and Pharmacy, 400012 Cluj-Napoca, Romania; dzdrenghea@yahoo.com (D.Z.); pop67dana@gmail.com (D.P.); 3 County Clinical Emergency Hospital of Sibiu, 2-4 Corneliu Coposu Str., 550245 Sibiu, Romania; 4 Research and Telemedicine Center for Neurological Diseases in Children, Pediatric Clinical Hospital Sibiu, 550166 Sibiu, Romania

**Keywords:** non-ST elevation acute coronary syndrome, myocardial infarction, unstable angina, atypical symptoms, gender differences

## Abstract

Clinical presentation is one of the factors that can influence how quickly a patient with an acute coronary syndrome is treated, particularly if it is atypical. The purposes of this study are to explore gender-related differences in patients presenting with non-ST elevation acute coronary syndromes (NSTEACS) from the perspective of a series of common risk factors as well as treatment strategies and to evaluate the prevalence of atypical clinical presentation of NSTEACS in the study group. In addition, we explored the differences between the two entities that define NSTEACS: unstable angina (UA) and non-ST elevation myocardial infarction (NSTEMI). We conducted a retrospective study by reviewing discharge documents of patients admitted in the cardiology department of the Clinical Rehabilitation Hospital in Cluj-Napoca with NSTEACS between January 2014 and December 2015. We retrieved demographic data, clinical presentation and history, laboratory tests, and coronary angiography records as well as the implemented treatment strategies. Women in the study group were more frequently hypertensive than men (89.5% vs. 75.4%; *p* = 0.043), had a higher mean serum HDL cholesterol value (43 vs. 38 mg/dL *p* = 0.022), were more frequently diagnosed with microvascular coronary heart disease (32% vs. 9.8%, *p* = 0.036), and were more often treated conservatively (49.1% vs. 30.8%, *p* = 0.038), while men were significantly more prone to smoking than women (30.8% vs. 14%, *p* = 0.028) and had higher mean serum creatinine (1.2 vs. 0.8 mg/dL; *p* = 0.022) and uric acid values (6.9 vs. 6.2 mg/dL; *p* = 0.048). Out of the 122 included patients, 109 had documented information regarding symptoms. The prevalence of atypical presentation was 4.6% (95% CI 0.7–8.5%). In our study group, patients with UA had a more frequent history of cardiovascular ischemic diseases (77.4% vs. 56.7%, *p* = 0.015), the mean value for BUN was higher in NSTEMI patients compared to patients with UA (47 vs. 39 mg/dL, *p* = 0.038) and NSTEMI patients more frequently received interventional treatment compared to patients with UA (60% vs. 41.9%; *p* = 0.046).

## 1. Introduction

Despite the recent progress in cardiovascular disease management, these types of illnesses are still some of the most frequently encountered worldwide [1]. Ischemia-related afflictions are the main culprits in this respect, with considerable effects on quality of life and mortality [2]. Acute coronary syndromes are some of the most impressive and threatening entities within this spectrum and are medical emergencies renowned for the importance of being timely managed [3,4]. Gibson C’s statement [5], “time is myocardium”, is one of the most evocative expressions portraying the relationship between treatment delay and adverse outcomes in acute myocardial infarction.

Clinical presentation is one of the factors that can influence how quickly a patient with an acute coronary syndrome is treated, particularly when this presentation is atypical. This refers both to the prehospital phase, when patients postpone seeking medical assistance due to not recognizing an acute coronary syndrome, as well as in-hospital door-to-treatment delays, even more so in situations where rapid diagnostic measures such as electrocardiography or bedside echocardiography may present unspecific results [6,7,8].

In addition, there is a keen contemporary interest related to the differences between genders concerning risk stratification, diagnosis, treatment strategies, and even recovery and rehabilitation of patients with acute coronary syndromes. These aspects have been highlighted by a recent exhaustive review of the literature performed by Mateo-Rodriguez et al. [9]. There is well-documented evidence that women with NSTEACS more frequently have atypical clinical presentations [10], have a greater delay to diagnosis and treatment [8], receive less invasive treatment and diagnosis procedures, and are less likely to receive guideline-indicated pharmacological and revascularization treatments [11]. Considerable efforts have been made to explain the mechanisms behind these differences. Factors such as psychological susceptibility, autonomic nervous system reactivity, and visceral innervation have been suggested to play a key role in the differences in pain characteristics in women presenting with ACS [12]. In addition, women may be susceptible to a different etiological profile of ACS due to increased vasospasm and more frequent spontaneous coronary artery dissection compared to men [13].

Therefore, studies reporting the differences between genders in patients with cardiovascular disease can always enhance the perspective of this yet unexplained matter.

The purposes of this study are to explore gender-related differences in patients presenting with NSTEACS from the perspective of a series of common risk factors as well as treatment strategies and to evaluate the prevalence of atypical clinical presentation of NSTEACS in the study group. In addition, we explored the differences between the two entities that define NSTEACS: UA and NSTEMI.

## 2. Materials and Methods

### 2.1. Study Design and Data Collection

We collected the data retrospectively by reviewing the discharge documents of patients admitted in the cardiology department of the Clinical Rehabilitation Hospital in Cluj-Napoca with NSTEACS between January 2014 and December 2015.

The inclusion criteria regarding the diagnosis of NSTEACS were verified by reviewing the discharge papers of patients admitted in the aforementioned timeframe. We included patients with the following diagnosis: 1. UA or NSTEMI diagnosed locally in the center where the study was conducted; 2. UA or NSTEMI diagnosed in a different center and referred to the center where the study was conducted for further investigation (mainly coronary angiography). The criteria for establishing the diagnosis of UA or NSTEMI were verified to be in accordance with those described in the most recently available European guidelines on the management of acute coronary syndromes in patients presenting without persistent ST-segment elevation [14].

For all the patients included, we retrieved the following data from the discharge documents: patient I.D., gender, age, personal history of atherosclerotic cardiovascular disease, presence of type 2 diabetes, arterial hypertension, smoking status, weight status, angina-related symptom characteristics, laboratory tests, and coronary angiography results as well as the treatment strategies utilized.

The presence of a personal history of atherosclerotic cardiovascular disease was considered in the presence of any of the following entities: history of coronary heart disease, cerebrovascular disease, peripheral artery disease. Sy et al. used a similar model for defining atherosclerosis-related diseases [15].

Weight status was approached as a dichotomous variable, where patients that had BMI ≥ 25 were considered overweight/obese and patients with a BMI < 25 were considered to have normal weight.

Typical and atypical angina was categorized post hoc (after reviewing the medical records of the included patients).

Typical angina-related symptom characteristics referred to chest discomfort that was with a precordial or retrosternal localization and of a constrictive/heavy character that could radiate to the neck/jaw/arms. This is in accordance with the descriptions found in the most recent ESC guidelines [14].

Patients described as having typical symptoms for NSTEACS, without explicit mention of pain characteristics, were included in this category as well. Patients who were diagnosed in a different center and had no description regarding NSTEACS symptoms were excluded from the calculation of atypical symptom prevalence.

Patients described as having atypical symptoms fell into one of the following categories: absence of pain on admission or mention of atypical pain characteristics in the medical records (such as chest pain indicative of a parietal nature that worsened with deep inspiration and upon palpation), or atypical pain character (for example, burning sensation).

Blood test results were also documented (total cholesterol, serum triglycerides, HDL cholesterol, LDL cholesterol, uric acid, BUN, serum creatinine).

The results from coronary angiography evaluations were reviewed and the number of affected vessels was documented. In this respect, patients were categorized as suffering from either 1,2,3-vessel disease (if one, two, or three of the following branches were affected: circumflex artery, interventricular artery, right coronary artery), left main disease, or microvascular disease if no macroscopic lesions were detected. The decision whether or not the involvement of a particular vessel was a significant contributor to coronary ischemia was based on the description of the interventional cardiology team in the postprocedural reports and the identification of lesions with >70% diameter stenosis or previous history of MI or coronary revascularization of the evaluated vessel. Similar categorical models and decision protocols for vessel involvement have been described in the literature [16,17].

According to the most invasive treatment approach utilized, patients were categorized as having received either conservative, interventional (balloon angioplasty or stent placement), or surgical treatment. This approach on categorizing treatment had a similar approach to Babatunde et al. [18].

### 2.2. Database Organization and Statistical Analysis

The correlations between variables were investigated using statistical tests in accordance with specific variable types, i.e., Chi-square test and Fisher exact test for qualitative variables and student *t*-test for quantitative variables. A significance level a under 0.05 was considered significant. The normal distribution of variables was investigated using the Shapiro–Wilk test and by analyzing kurtosis and skewness. Confidence intervals were constructed using a 95% confidence level. The 95% confidence interval for atypical symptom prevalence was determined by post sample collection marginal error calculation $E=z\sqrt{pq/n}$, where *n* is the sample size, *p* is the proportion of patients with atypical symptoms, and *q* is the proportion of patients with typical symptoms.

Data visualization and analysis were conducted using Microsoft Excel^®^ and SPSS^®^ (v. 21.0.0.0).

### 2.3. Sample Size

In order to calculate sample size, we used the formula $n=\frac{z^{2}p\left(1-p\right)}{d^{2}}$, where *n* = sample size; *z* = 1,96, the z value for 95% confidence interval; *p* = estimated prevalence of a target dichotomous parameter—in our case, the presence of atypical symptoms; *d* = margin of error accepted (5% in our case). Similarly to Chowdhury et al. [19], the value of *p* was derived from a relevant study examining the prevalence of our researched dichotomous variable. We found a study conducted by Zdzienicka J. et al. [20], which included 2382 patients from 29 hospitals and found a mean prevalence of 6.4% of atypical symptoms in NSTEMI to be adequate in this respect. Thus, for *p* = 0.064, *n* = 93 (rounded up). Our study included 122 patients, 109 of which had descriptions of their clinical presentation.

## 3. Results

### 3.1. Demographic Data

A total of 122 patients met the inclusion criteria—57 women and 65 men—with a mean age of 65.20 years (33–85 years), which were normally distributed according to age (p=0.075). There were 60 patients diagnosed with NSTEMI (26 women, 34 men, mean age 65.13 years) and 62 diagnosed with UA (31 women, 31 men, mean age 65, 26 years). The two diagnostic groups also presented a normal distribution according to age (NSTEMI, p=0.515; UA, p=0.172). We found no significant differences between the mean ages of patients with NSTEMI and UA (*p* = 0.951), the mean ages of patient genders (66.18 years for women and 64.34 years for men, p=0.366), or between gender and UA/NSTEMI diagnosis (p=0.461).

### 3.2. Clinical Presentation

Of the included patients, 109 had documented information regarding their symptoms. Atypical symptoms were presented by five patients representing a percentage of 4.6% (95% CI 0.7–8.5%); three of them were diagnosed with UA (4.9% of UA patients) and two patients were diagnosed with NSTEMI (4.2% of NSTEMI patients).

### 3.3. Risk Factors

The distribution of the risk factors within the study group is presented in Table 1.

AtsHx refers to a history of atherosclerotic cardiovascular disease, as previously defined. Patients with UA had significantly more frequent AtsHx when compared to patients with NSTEMI.

Women in the study group were more frequently hypertensive than men, while men were significantly more prone to smoking than women.

Data regarding serum values in the study group corresponding to the lipid profile and circulating uric acid are presented in Table 2.

The mean value for BUN was higher in NSTEMI patients when compared to patients with UA.

With regard to gender-related differences, women had a statistically significant higher mean HDL-C value, while men had higher circulating creatinine and uric acid values.

### 3.4. Coronarography Results

A total of 111 patients underwent coronary angiography investigations. The results are presented in Table 3.

In the study group, microvascular disease was significantly more frequent amongst women than men.

### 3.5. Treatment Strategies

The employed or recommended treatment strategies utilized within the study group are presented in Table 4.

In the study group, patients with NSTEMI were more likely to receive interventional treatment.

Women were significantly more likely to undergo conservative treatment compared to men.

## 4. Discussion

The purposes of this study are to report our findings concerning gender-related differences in patients with NSTEACS in the ongoing pursuit of describing these circumstances and to assess the prevalence of atypical symptoms in our study group. In addition, we aimed to describe the differences we found between patients with UA and NSTEMI. The data collected included information regarding demographic distribution, risk factors, and therapeutic approaches.

There is a keen interest in attempting to describe the causality behind the striking differences between genders in cardiovascular disease. Consistent efforts have been made in the literature to establish the connection between the underlying mechanisms concerning vascular impairment and the particular characteristics of female cardiovascular patients. The most mentioned pathways include pathological vasoreactivity such as spasm and endothelial dysfunction, which is more frequent in women, with significant adverse implications on microvascular physiology [21]. In our study, the majority of patients with microvascular disease were women. This aspect is also supported in other studies [22,23,24,25,26]. The triad of anginal pain, effort-dependent ischemia detected on ECG, and normal angiographic coronary arteries define the so-called “X-coronary syndrome” [27] and is equivalent to microvascular ischemic heart disease [28]. Assessment of coronary microvascular function by Doppler examination coronary flow velocity reserve (CFVR) of the left anterior descending coronary has shown that CVFR impairment is found in a substantial proportion of women with microvascular disease, with a weak association to classical cardiovascular risk factors, implying possible distinct pathways leading to microvascular disease and angina [29]. The particular array defining the cardiovascular status of women is also age-dependent. Women develop cardiovascular disease later than men and have a different distribution of risk factors, being less likely to smoke and with higher levels of circulating HDL cholesterol as protective factors but more prone to hypertension as shown by some studies [28,30]. HDL cholesterol has a well-known protective influence on the development of atherosclerosis by mediating cellular cholesterol metabolism, apoptosis, vascular tonus, inflammation and oxidative stress, platelet activation, and glucose metabolism. Additionally, certain parameters showing differences between genders, such as circulating uric acid and its implications in endothelial dysfunction, inflammation, oxidative stress, and arterial stiffness may further widen the gap between genders regarding the mechanisms behind vascular impairment [31,32]. Our study has found similar results in characterizing the typical risk clusters and parameters of women versus men with cardiovascular disease. In particular, women in our study were less prone to be smokers, had a higher mean level of HDL-cholesterol and a lower mean level of circulating uric acid when compared to men but were more frequently hypertensive.

Clinical presentation of angina is also subject to significant gender differences, as suggested by several studies showing that women are more susceptible to atypical anginal symptoms [14,20].

One further aspect to be considered is that in this study group, women were more frequently treated conservatively compared to men. The idea that women with coronary heart disease present more often with unspecific symptoms and frequently receive less invasive diagnostic and therapeutic management requires particular attention in the clinical setting. This is in agreement with the findings published by Jackson et al. [11], where there is even a suggestion of adapting the current guidelines to gender-specific differences.

The aforementioned aspects lead to the hypothesis of the possible existence of a subgroup of patients in which the diagnosis of unstable angina, though present, cannot be ascertained based on either clinical, electrocardiographic (i.e., nonspecific ECG signs in NSTEACS), biomarker, or angiographic characteristics. Such patients are essentially the intersecting area between (1) NSTEACS patients with unspecific ECG modifications, (2) UA patients (with no elevated cardiac troponins), (3) atypical clinical presentation of NSTEACS, and (4) no detectable hemodynamically significant lesions on coronary angiography (microvascular disease). These patients may be a challenge when deciding on the optimal course of action.

Of the 109 patients with NSTEACS with documented symptom characteristics, five had an atypical clinical presentation (4,6%; 95% CI 0.7–8.5%). The prevalence of atypical symptoms in NSTEACS has been extensively studied. The data in the literature are subject to extensive variability, thus allowing for no general consensus regarding this parameter. One study, which is also quoted by the latest ESC guidelines on the management of NSTEACS [14], included 4167 patients admitted to 22 hospitals in Alabama, USA between 1993 and 1995 and found a prevalence of no less than 51.7% of atypical symptoms in patients presenting with unstable angina pectoris, according to their definition of typical and atypical symptoms [33].

Another study, based on the data obtained from the Framingham cohort, found that over one-quarter of myocardial infarctions within a time frame of about 30 years were diagnosed incidentally during routine ECG exams performed once every two years. Half of these myocardial infarctions were completely silent [34].

The definitions of typical or atypical symptoms are probably one of the main issues regarding the great variability of the results concerning the prevalence of atypical clinical presentation. In our study, we attempted an approach centered on the findings of clinicians during the acute phases of NSTEACS. We considered that if the atypical character of symptoms were sufficiently important to be documented in the discharge papers, it is reasonable to assume that the clinical presentation had an impact not necessarily on the location, character, or duration of chest pain, but rather on an important overall attribute of symptomatology, namely, clinical relevance. By exhaustively reviewing the discharge documents of the included patients, instead of predefining what would be considered typical or atypical symptoms, we relied on the clinical rationale of the descriptions of these records and based the decision of assigning patients to either the typical or atypical symptoms group accordingly. The results of our study are comparable to the one conducted by Zdzienicka J. et al. [20]. In essence, the post hoc structuring of typical versus atypical pain criteria is, however, an element of novelty in this study and prompts the development of more standardized approaches when defining clinical presentation, ideally in a prospective manner.

One of the implications of this study concerns potential risk factor control and screening efforts regarding ischemic cardiovascular pathology. Patients with UA were more likely to have relevant ischemic cardiovascular disease history. From a different point of view, this would imply that for a large proportion of patients with NSTEACS, the first manifestation of an ischemic cardiovascular disease was a myocardial infarction.

Patients with NSTEMI were more likely to receive interventional treatment, possibly due to the more frequent identification of a culprit lesion during coronary angiography.

### 4.1. Study Limitations

The conductance of a retrospective study is known to be prone to a series of well-known possible errors. Our study, however, brings interesting data concerning the distinction between typical and atypical symptoms. A prospective study based on the same principles as our current effort may provide superior results. The results of our study are in agreement with the current literature.

A further aspect to consider that may have affected the quality of this study concerns the way the sample of patients was constructed. Firstly, the patients admitted in the cardiology ward of the Clinical Rehabilitation Hospital in Cluj-Napoca mostly originate from a relatively small geographic area and present mostly Caucasian features. Thus, the obtained results may not be ideal for extrapolation to the general population. Secondly, this was a single-center study. A multicentric approach could provide more significant data in this respect.

Sample size may be regarded as a limitation for our study. The method we used to calculate the necessary sample size, however, has been successfully employed previously in the literature [19] and should assert the validity of the data reported in this study.

### 4.2. Future Directions

Our results could make a significant contribution to further prospective large studies to obtain additional gender-specific and clinical data on NSTEACS patients. Conducted for a sufficiently long period of time and upon a large sample of patients, future research approaches might show promise regarding gender-based risk evaluation for NSTEACS patients. In addition, further testing of specific biomarkers such as laboratory (CRP, procalcitonin, neutrophil-to-lymphocyte ratio), electrocardiographic, and imaging markers (echocardiography, MRI) could enhance these results.

## 5. Conclusions

Based on the information presented, it may be safe to conclude that, despite the limitations regarding data collection and sample size, the results obtained in this study can be regarded as relevant. The methodology presented could aid in the design of further studies exploring gender-specific characteristics of patients with NSTEACS. The ultimate goal in this respect would be to avoid potential delays in providing them with optimal treatment, particularly when presenting with atypical symptoms. 

## Figures and Tables

**Table 1:** Risk factor distribution and Chi-square test results.

Risk Factor	Total	NSTEMI	UA	*p*-Value	Women	Men	*p*-Value
AtsHx	82	34	48	0.015	38	44	0.904
	67.2%	56.7%	77.4%		66.7%	67.7%	
Type 2 DM	44	21	23	0.809	25	19	0.093
	36.1%	35.0%	37.1%		43.9%	29.2%	
Hypertension	100	50	50	0.699	51	49	**0.043**
	82%	83.3%	80.6%		89.5%	75.4%	
Smoking	28	13	15	0.740	8	20	**0.028**
	23%	21.7%	24.2%		14%	30.8%	
Overweight	31	16	15	0.754	18	13	0.143
	25.4%	26.7%	24.2%		31.6%	20%	

**Table 2:** Laboratory tests and student *t*-test *p*-values for independent variables.

Bood Test (mg/dL)	UA		NSTEMI		*p*-Value	Women		Men		*p*-Value
	Mean	StdDEV	Mean	StdDEV		Mean	StdDEV	Mean	StdDEV	
TGL	183	188	151	70	0.232	178	193	158	83	0.463
TChol	187	64	176	40	0.275	192	64	173	41	0.055
LDL-C	111	41	108	36	0.638	115	42	103	35	0.092
HDL-C	41	13	39	9	0.265	43	12	38	10	**0.022**
Uric Acid	6.5	1.7	6.6	2	0.834	6.2	2	6.9	1.6	**0.048**
BUN	39	13	47	24	**0.038**	40	19	45	20	0.218
Creatinine	0.9	0.3	1.1	1	0.124	0.8	0.3	1.2	1	**0.022**

TGL—Serum Triglycerides, TChol—Total Cholesterol, LDL-C—LDL Cholesterol, HDL-C—HDL Cholesterol, BUN—Blood urea nitrogen.

**Table 3:** Coronary angiography results and Chi-square test results.

Disease Type	Total	NSTEMI	UA	*p*-Value	Women	Men	*p* Value
3-vessel	30	14	16	0.405	12	18	0.516
	27%	23.7%	30.8%		24%	29.5%	
2-vessel	30	17	13	0.652	10	20	0.131
	27%	28.8%	25%		20%	32.8%	
1-vessel	24	15	9	0.299	10	14	0.707
	21.6%	25.4%	17.3%		20%	22.9%	
Left Main	5	3	2	0.098	2	3	0.817
	4.5%	5%	3.8%		4%	4.9%	
Microvascular	22	10	12	0.419	16	6	**0.036**
	19.8%	16.9%	23%		32%	9.8%	

**Table 4:** Treatment options and Chi-square test results.

Treatment	Total	NSTEMI	UA	*p*-Value	Women	Men	*p*-Value
Conservative	48	21	27	0.334	28	20	**0.038**
	39.3%	35%	43.5%		49.1%	30.8%	
Interventional	62	36	26	**0.046**	25	37	0.149
	50.8%	60%	41.9%		43.9%	56.9%	
Surgical	12	3	9	0.078	4	8	0.327
	9.8%	5%	14.5%		7%	12.3%	

## Data Availability

The data presented in this study are available on request due to privacy restrictions.
